# Color plumage polymorphism and predator mimicry in brood parasites

**DOI:** 10.1186/1742-9994-10-25

**Published:** 2013-05-10

**Authors:** Alfréd Trnka, Tomáš Grim

**Affiliations:** 1 Department of Biology, University of Trnava, Priemyselná 4, Trnava SK-918 43, Slovakia; 2 Department of Zoology and Laboratory of Ornithology, Palacký University, 17. listopadu 50, Olomouc CZ-771 46, Czech Republic

**Keywords:** Brood parasitism, Frequency-dependent selection, Mimic, Model, Polymorphism

## Abstract

**Background:**

Plumage polymorphism may evolve during coevolution between brood parasites and their hosts if rare morph(s), by contravening host search image, evade host recognition systems better than common variant(s). Females of the parasitic common cuckoo (*Cuculus canorus*) are a classic example of discrete color polymorphism: gray females supposedly mimic the sparrowhawk (*Accipiter nisus*), while rufous females are believed to mimic the kestrel (*Falco tinnunculus*). Despite many studies on host responses to adult cuckoos comprehensive tests of the “hawk mimicry” and “kestrel mimicry” hypotheses are lacking so far.

**Results:**

We tested these hypotheses by examining host responses to stuffed dummies of the sparrowhawk, kestrel, cuckoo and the innocuous turtle dove (*Streptopelia turtur*) as a control at the nest. Our experimental data from an aggressive cuckoo host, the great reed warbler (*Acrocephalus arundinaceus*), showed low effectiveness of cuckoo-predator mimicry against more aggressive hosts regardless of the type of model and the degree of perfection of the mimic. Specifically, warblers discriminated gray cuckoos from sparrowhawks but did not discriminate rufous cuckoos from kestrels. However, both gray and rufous cuckoos were attacked vigorously and much more than control doves. The ratio of aggression to gray vs. rufous cuckoo was very similar to the ratio between frequencies of gray vs. rufous cuckoo morphs in our study population.

**Conclusions:**

Overall, our data combined with previous results from other localities suggest polymorphism dynamics are not strongly affected by local predator model frequencies. Instead, hosts responses and discrimination abilities are proportional, other things being equal, to the frequency with which hosts encounter various cuckoo morphs near their nests. This suggests that female cuckoo polymorphism is a counter-adaptation to thwart a specific host adaptation, namely an ability to not be fooled by predator mimicry. We hypothesize the dangerousness of a particular model predator (sparrowhawks are more dangerous to adult birds than kestrels) may be another important factor responsible for better discrimination between the gray cuckoo and its model rather than between the rufous cuckoo and its model. We also provide a review of relevant existing literature, detailed discussion of plumage polymorphism in cuckoos, methodological recommendations and new ideas for future work.

## Introduction

Polymorphism in external color, i.e. coexistence of two or more different color morphs in the same population of a species, is a widespread phenomenon in many animal taxa [1]. In birds, color polymorphism involves 3.5% of all bird species, most frequently occurring in birds of prey [2], owls and nightjars [2,3], and cuckoos [3]. All polymorphic cuckoos (12% of all cuckoo species [3]) are brood parasites [4]. The best known example is the iconic Old World parasite, the common cuckoo (*Cuculus canorus*; hereafter: cuckoo). Males are monomorphic (gray), but females come in two discrete morphs: they either look roughly the same as the male or come in a very different rufous color. The frequency of the two morphs (morph-ratio) varies dramatically from sites where rufous females numerically dominate over gray females [5] to sites where rufous females are virtually lacking [6]. This is perhaps because of negative-frequency dependent selection: hosts may better discriminate the more common morph while the rarer morph gains an advantage simply by being less often encountered (and, by implication, recognized and remembered as a parasite) by hosts [4-6].

Recent studies suggest female coloration in cuckoos may be the result of predator mimicry, showing both Batesian and aggressive mimicry aspects, with dangerous predators being models and brood parasite hosts being dupes [7-9]. Gray females are thought to mimic sparrowhawks (*Accipiter nisus*; “hawk mimicry” hypothesis; [6,7]) while rufous females are thought to mimic kestrels (*Falco tinnunculus*; “kestrel mimicry” hypothesis; [10]). Cuckoos may gain fitness benefits from predator resemblance due to easier access to host nests (i.e., avoiding attacks by hosts; alternatively, they may benefit from cryptic aspects of their plumage [11]). In an analogy to egg mimicry the predator mimicry hypothesis predicts the mimetic cuckoo morph should elicit the same response as its model. Therefore, considering that the receiver responses maintaining the plumage polymorphism are the joint result of frequency-dependent selection involving both mimic and model, the success of respective morphs should additionally be affected by the frequencies of their respective models [5].

To date, only two experimental studies have focused on the phenomenon of adult parasite plumage polymorphism (for comparative studies see [4,11,12]). Honza et al. [5] supported the hypothesis that the geographical variation in the abundance of gray vs. rufous cuckoo morph may affect host aggression, but did not provide a direct test of the predator mimicry hypothesis. However, in none of the two studied populations did hosts clearly discriminate (i.e., contact attacks) between the two color morphs (but this may have been caused by methodological constraints, see Table 1 and Discussion in the present work). Thorogood and Davies [6] showed cuckoo color-polymorphism may be a parasite adaptation against socially transmitted learned host defenses. Specifically, reed warbler (*Acrocephalus scirpaceus*) hosts increased their mobbing of cuckoo dummies only when the same color-morph dummy was recently mobbed by their neighbors (but did not change their aggression levels when the other color-morph dummy was previously attacked by warbler neighbors). However, during the past 10–20 years almost exclusively gray cuckoo females have occurred in both the UK and Czech Republic and there is no evidence for changes in morph ratio over that period in any of those localities ([13], references above and M. Honza pers. comm.). This raises an intriguing question: why are the numbers of rufous cuckoos not increasing as predicted by the negative-frequency dependent selection hypothesis? One possible solution to this paradox is that local frequencies of predator models affect host responses: where there are more kestrels than sparrowhawks (e.g., in the open country), it might be more beneficial for cuckoos to mimic kestrels because hosts are less familiar with the other predator model [10]. The answer may also lie in imperfect kestrel mimicry [14]. However, although the quality and effectiveness of hawk mimicry has already been tested experimentally by comparing host and non-host responses to gray cuckoos vs. sparrowhawks [7,8,15], no study has so far directly tested for kestrel mimicry.

**Table 1:** **Great reed warbler responses to experimental dummy dyads (see Methods and Figure **1**)**

**Experiment**	**N**	**Preference**	**Continuous response**		**Categorical response**	
		**(%)**	**Z**	**P**	**Z**	**P**
Specific recognition						
**Gray cuckoo**–Dove	18	100	85.5	<0.0001	45.5	0.0002
**Rufous cuckoo**–Dove	16	94	66.0	<0.0001	7.5	0.06
**Kestrel**–Dove	17	88	67.5	0.0005	7.5	0.06
**Sparrowhawk**–Dove	14	93	46.5	0.0016	5.0	0.13
Two morphs comparison						
**Gray cuckoo**–Rufous cuckoo	18	72	48.0	0.035	0.0	1.00
Mimicry						
**Gray cuckoo**–Sparrowhawk	23	96	135.0	<0.0001	7.5	0.06
Rufous cuckoo–**Kestrel**	20	70	35.0	0.20	0.0	1.00

Dummies within dyads that were mobbed more than their paired dummy are in bold. “Preference” is the percentage of warbler pairs that more strongly responded to the more attacked dummy (in bold) than to the paired dummy. The “Specific recognition” set of experiments asked “Do warblers recognize dangerous enemies near the nest specifically?” by comparing responses to cuckoos/predators with responses to harmless control turtle dove. The “Mimicry” set of experiments asked “Do gray cuckoos mimic sparrowhawks and do rufous cuckoos mimic kestrels?” N = number of host pairs. Responses were measured either as number of contact attacks (Continuous response) or re-coded as presence vs. absence of attacks (Categorical response). See Discussion for rationale behind and implications of categorical re-coding. Differences tested with Wilcoxon sign-rank tests.

Therefore, one of the most fundamental empirical questions remains untested: do hosts indeed mistake *both* cuckoo color morphs for their respective supposed models? This requires testing host responses towards the gray morph and rufous morph cuckoos, sparrowhawks, kestrels and a control simultaneously in one host species and under a consistent experimental design (as argued by [9]). Here, for the first time, we simultaneously tested the quality of gray cuckoo-hawk and rufous cuckoo-kestrel mimicry in one of the major cuckoo hosts, the great reed warbler (*Acrocephalus arundinaceus*; hereafter: warbler). This seems to be an especially suitable species for such a test because warblers are aggressive and clearly recognize the cuckoo as a special enemy in our study population [15,16].

In our study site gray cuckoos are more common than the rufous ones; still, the rufous morph is much more common at our study site (40%, Results) than in the UK site where the evidence for frequency-dependence was obtained (~1%, see [6]). Additionally, sparrowhawks are present at the UK study site (N. B. Davies, pers. comm.) whereas they are absent at our study site (Results). These differences provide a good opportunity to test host discrimination abilities in an ecological context differing in relevant parameters (morph-ratio, model-ratio) from previously studied localities (see also [5]). Thus, based on predictions of the frequency-dependent selection hypothesis and findings on social learning [6], we should expect our warbler population will discriminate better between gray cuckoo morph and its respective model (sparrowhawk) than between rufous cuckoo morph and its respective model (kestrel). In line with findings of Honza et al. [5], we predicted that warblers will behave more aggressively towards the more common gray cuckoo morph than towards the rarer rufous cuckoo morph.

A necessary pre-requisite of any enemy discrimination study is to first establish that hosts show specific recognition of enemies near the nest, i.e., their responses are not an unspecific by-product of generalized nest defense against any – even harmless – intruders [17,18]. Therefore, we first tested whether warblers indeed recognize gray cuckoo, rufous cuckoo, sparrowhawk and kestrel as special enemies. We tested this by comparison of host responses to cuckoo/predator dummies paired in experimental dyads (see Methods) with a harmless control, the turtle dove (*Streptopelia turtur*) (see also [15]). Second, we tested whether warblers would be more aggressive towards gray or rufous morph (see also [5,6]). Finally, we tested whether warblers discriminate between the gray cuckoo and its model (the sparrowhawk) better than between the rufous cuckoo and its model (the kestrel).

## Results

### Frequency of mimetic morphs and models

The numbers of females of the two color morphs were 5 gray and 3 rufous in 2012 in the study area. This ratio (~60% vs. ~40%) is further supported by more frequent sightings of gray than rufous cuckoo females in previous field seasons (AT unpubl. data). Sparrowhawks were not observed in 2012 or during previously observed breeding seasons (see also [16]). In contrast, kestrels have been regularly breeding in the vicinity of fishponds (1–2 pairs in 2012 and previous breeding seasons). These patterns are further supported by the fact that the sparrowhawk shows a distributional gap during the breeding season in south Slovakia (see p. 188 in [19]), whereas the kestrel shows a continuous breeding distribution in the same mapping quadrats, including our study site (see p. 208 in [19]).

### Responses to dummies

**Figure 1:**
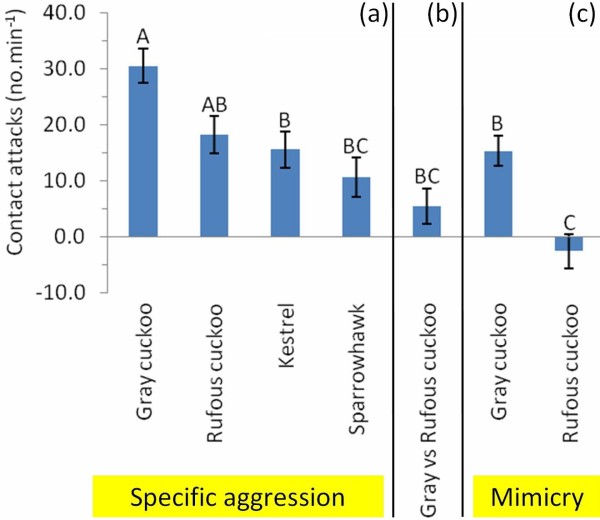
**Three sets of experiments to test for enemy recognition and mimicry hypotheses (see Introduction).** Response (aggression measured as number of contact attacks per 1 min.) is presented as a *difference* (mean ± s.e.) between aggression levels towards two dummies within a simultaneously presented dyad (see Methods). For sample sizes see Table 1. (**a**) “Specific aggression” is the aggression to cuckoo/predator dummy minus baseline aggression to control (dove), i.e., specific response to the dangerous enemy above the background aggression shown to any, even innocuous, intruders near the nest (see [18]). (**b**) Difference between responses to paired gray vs. rufous cuckoo female morph. (**c**) “Mimicry” is the aggression towards a particular cuckoo morph minus aggression towards its respective model (i.e., sparrowhawk for gray morph, kestrel for rufous morph). Different letters indicate statistical differences between groups according to GLMM (Tukey HSD, α = 0.05).

Simple comparisons of responses within experimental dyads showed warblers clearly recognized all cuckoo and predator dummies from a harmless control dove (Table 1, see also Figure 1a and statistical results of other analyses below). In 88–100% of “specific recognition” experiments, warblers responded more strongly to the dangerous intruders compared to a harmless control. Responses to gray cuckoos were significantly stronger than those to paired rufous cuckoos (Table 1, Figure 1b). Finally, warblers clearly discriminated gray cuckoos from sparrowhawks, but failed to discriminate rufous cuckoos from kestrels (Table 1, Figure 1c).

Further comprehensive tests (GLMM) showed that additional factors explained variation in warbler aggressive behavior, but did not affect the general conclusions (Table 2). Aggression decreased linearly throughout the breeding season. In approx. half of experiments (52%, n = 126) female warblers started the attack. Males rarely (10%) attacked before females did. In other cases (38%) both sexes attacked simultaneously. Experiments when both parents launched the attack simultaneously showed higher aggression than those where only the male or only the female started to attack before the other partner (Tukey-Kramer HSD tests: “both parents” differed from “female first”, “male first” did not differ from either of the two categories, α = 0.05). Host arrival direction had a weak effect in the final model and no effect according to a post-hoc test (Tukey-Kramer HSD: no significant differences at α = 0.05). Post-hoc testing (Tukey-Kramer HSD) showed that, controlling for other factors (Table 2), the responses decreased from gray cuckoos, through kestrels, rufous cuckoos, sparrowhawks to doves. Critically, responses to the gray cuckoo were stronger than those to the sparrowhawk whereas responses to the rufous cuckoo did not differ from those to the kestrel. Warblers attacked gray cuckoos more than rufous cuckoos; thus, analyses that statistically take into account possible confounders (Table 2) corroborate conclusions based on simple non-parametric paired comparisons (Table 1).

**Table 2:** Predictors of great reed warbler responses to dummy presentations at their nests

**Minimal adequate model**	**df**	**F**	**p**	**Estimate (S.E.)**
Intercept				17.23 (0.88)
Dummy type (dove)^a^	4,220.20	45.85	< 0.0001	gray cuckoo: 9.63 (1.21)
				kestrel: 5.50 (1.44)
				rufous cuckoo: 3.82 (1.26)
				sparrowhawk: −6.61 (1.45)
First attacking sex (male)^a^	2,161.50	7.85	0.0006	female: −1.69 (0.97)
				both: 3.59 (1.04)
Experimental date – linear	1,73.06	10.71	0.002	−0.32 (0.10)
Host arrival direction (focal)^a^	2,173.80	4.23	0.02	central: −2.72 (0.93)
				opposite: 1.33 (1.04)
**Removed predictors**				
Final clutch size	1, 76.30	0.26	0.61	−0.70 (1.36)
Experimental date – quadratic	1, 54.42	2.10	0.15	0.01 (0.01)

Responses were measured as the number of contact attacks per first minute of the experiment (see Methods). Results of the general linear mixed models (GLMM).

^a^ Reference levels of categorical variables are given in brackets.

Further, we repeated the analyses but instead of response per dummy (above) we used difference in aggression within dummy dyad (therefore the predictor “dummy” was changed for “experiment type”). All potential covariates were non-significant and the final model contained only the highly significant effect of experiment type (F_6,113.1_ = 11.46, P < 0.0001; Figure 1). After controlling for “background” aggression to the control dummy (dove), the host “specific” aggression was strongest to the gray cuckoo and weakest to the sparrowhawk (Figure 1a). Differential aggression to two cuckoo morphs was positive and significantly different from zero (i.e., higher response to gray vs. simultaneously presented rufous morph; Figure 1b). Specific aggression to the gray cuckoo (vs. its model sparrowhawk) was positive and significantly different from zero whereas specific aggression to the rufous cuckoo (vs. its model kestrel) was negative, small and not significantly different from zero (Figure 1c).

When we re-coded continual responses on a categorical scale, most of the significant effects detected by the analyses of continuous responses disappeared (Table 1). This was because each dummy within a dyad at a majority of nests received at least one contact attack. See Discussion for the implication of this result.

## Discussion

Plumage polymorphism is a conspicuous aspect of brood parasitic cuckoos, well known to both scientists and laymen. However, this fascinating phenomenon remained virtually unstudied until recently [5,6,11,12]. Here, we extended this pioneering work by experimentally testing several hypotheses on cuckoo predator mimicry, including the first empirical test of the kestrel mimicry hypothesis.

As predicted, great reed warblers showed different discrimination abilities and aggressiveness towards gray and rufous cuckoo morphs in our study population. They clearly discriminated gray cuckoos from sparrowhawks, but failed to discriminate between rufous cuckoos and kestrels. Generally, however, warblers attacked both gray and rufous cuckoos near their nests. Thus, our results support previous findings suggesting the low effectiveness of cuckoo-predator mimicry against more aggressive hosts (like great reed warblers), regardless of the type of model (i.e., sparrowhawk or kestrel) and the degree of perfection of the mimic (i.e., similarity between the model and the mimic; [15,20-22]). That great reed warbler attacks can have fatal consequences for cuckoos was supported by a rufous cuckoo female being found killed under the warbler nest in our study site (AT unpubl. data, for more evidence of cuckoos being killed by great reed warblers, see [23,24]). Importantly, gray cuckoos were attacked more frequently than rufous ones and the result remained the same when we analyzed raw host responses or responses corrected for background aggression.

The observed levels of warblers’ species-specific aggression were in line with the frequency of the two cuckoo morphs. Specifically, aggression towards the gray cuckoo was ~1.7 times higher than specific aggression towards the rufous cuckoo (Figure 1a). This specific aggression ratio was very close to the ratio between frequencies of gray vs. rufous cuckoo morphs in our study population (~1.7; the ratio for overall uncorrected aggression would be similar: ~1.3, see Table 1). This similarity would make biological sense if host responses and discrimination abilities are directly proportional to the frequency with which they encounter various cuckoo morphs near their nests (as found by [6]). That warblers recognized the gray morph as a special enemy, but did not specifically recognize the rufous morph parallels findings that cuckoo hosts recognize the cuckoo as a special enemy in highly parasitized populations, but fail to do so in less parasitized populations [5,25].

The abundance ratio of predator models (sparrowhawks and kestrels), on the other hand, correlated negatively with the abundance ratio of cuckoo morphs. Warblers showed higher aggression towards and better discrimination of the more common (gray) cuckoo morph mimicking model (sparrowhawk) that was absent from the study area. In contrast, they showed lower aggression towards and poorer discrimination of the less common (rufous) cuckoo morph that mimicked the more common model (kestrel). Hence, these data support the previous results [5,6] about the crucial importance of relative morph ratios. At the same time, our results suggest the frequency of models (sparrowhawk vs. kestrel) does not override the morph ratios as a driver of host morph-specific responses on a small geographical scale.

Thus, the patterns of warbler aggression and discrimination abilities are generally in line with the view that negative frequency-dependent selection stabilizes enemy polymorphism. Specifically, after controlling for background aggression (sensu [18]) towards the control dummy (dove), warblers showed specific discrimination of all cuckoo morphs and their supposed models (Figure 1a). Differential aggression between gray and rufous cuckoo was positive and significantly larger than zero (Figure 1b). Incremental specific aggression towards the gray cuckoo (above background aggression towards its model, i.e., sparrowhawk) was large and significantly differed from zero. In contrast, incremental specific aggression towards the rufous cuckoo (above background aggression to its model, i.e., kestrel) was small, negative and not significantly different from zero (Figure 1c). Our conclusion remained the same when we took possible confounding variables into account (Table 2).

Further tentative support for the frequency-dependence scenario comes from the relationship between morph-ratios and relative host aggression towards morphs across geographical replicates (Table 3). Despite different model host species, study design and measures of host responses, there is a general tendency (although statistically sometimes non-significant) in the UK, Hungary and Slovakia (this study) that the locally more common cuckoo morph is mobbed more than the rarer morph [5,6, this study]. However, in the Czech Republic the opposite non-significant tendency was observed probably due to a low overall parasitism rate [5]; alternatively, this result may be a methodological artifact (see below). In contrast, hosts strongly mobbed cuckoo morphs mimicking models that were absent from the particular study site (e.g., almost all great reed warblers attacked the gray cuckoo morph in Hungary where sparrowhawks are absent, Table 3). These suggestive patterns therefore highlight the need for large-scale work across various populations with independently varying frequencies of models (predators) and mimics (cuckoo morphs; see also [9]).

**Table 3:** Overview of experimental studies of plumage polymorphism in cuckoos

**Host**	**Locality**	**Model**		**Mimic**		**Aggression**	
		**Species**	**Presence**	**Morph**	**Frequency**	**Calls**	**Attacks**
RW	UK^a^	Sparrowhawk	1	Gray	99	50	–
		Kestrel	1	Rufous	1	38	–
GRW	CZ	Sparrowhawk	1	Gray	90	65	17
		Kestrel	0	Rufous	10	79	21
GRW	SK	Sparrowhawk	0	Gray	60	–	76
		Kestrel	1	Rufous	40	–	24
GRW	HU	Sparrowhawk	0	Gray	40	93	50
		Kestrel	1	Rufous	60	97	73

^a^ Estimated according to information from [5]: 20 years data, each year ~10 cuckoo females, total “only one rufous female in two of the years”, i.e., 2/200 = 1%.

Localities were situated in United Kingdom (UK; [5]), Czech Republic (CZ; [4]), Hungary (HU; [4]) and Slovakia (SK; this study). Data are ordered from gray-morph dominated localities to those where the rufous-morph is more common. The presence (0/1) of models for each cuckoo morph (N. B. Davies, M. Honza, pers. comm.), frequency of each cuckoo morph (%), and responses of reed warblers (RW) and great reed warblers (GRW) to each morph are shown. Responses were measured either as proportion of host pairs that performed particular behavior (UK, CZ, HU), or as a proportion of contact attacks from the gray–vs.–rufous cuckoo paired treatment (SK, this study; tested pairs attacked each dummy at least once, thus, eliminating information potential of categorical assessment of host behavior, see also Table 1). See Discussion for details.

Caution is needed when interpreting current morph ratios as a potential explanation of current host enemy discrimination behavior. This is because we do not know the time frame over which the frequency of morphs or models changes. Hence, it is possible that current morph ratios reflect past rather than current selection (i.e., evolutionary lag). However, experiments of Thorogood and Davies [5] provide direct evidence that current (i.e., experimentally manipulated) morph ratios (i.e., probability of observing gray vs. rufous cuckoo near a host nest) affect host behavior. Still, it will be interesting to examine morph ratio vs. host behavior dynamics over longer time scales both theoretically (modeling approach) and empirically (by replicating enemy discrimination experiments in the same study sites after mimic and/or model ratios changed).

Nevertheless, the most striking result of the present study was that warblers exhibited a strong ability to discriminate between gray cuckoos and sparrowhawks whereas they were completely unable to discriminate between rufous cuckoos and kestrels. This could indicate that the rufous cuckoo is a better predator imitator compared to the gray cuckoo and that kestrel mimicry (if it is indeed mimicry, see [12]) is more perfect than hawk mimicry. In light of the findings mentioned above, a more plausible explanation is that warblers discriminated better between the gray and rufous cuckoos and their respective models because gray cuckoos dominated the study area and thus hosts had more opportunities to learn (through direct experiences or social learning [6,26]) to discriminate between them. This is in line with the hypothesis that the mimic-model discrimination ability of hosts depends on the local actual cuckoo morph ratio [6]. Thus, female cuckoo polymorphism may be a counter-adaptation to thwart a specific host adaptation, namely an ability to not be fooled by predator mimicry.

However, there is an additional factor that may explain why aggression towards the rufous morph was smaller than that towards the gray morph. The overall danger posed by a sparrowhawk is quite different from a kestrel. Sparrowhawks eat mainly birds whereas kestrels eat mainly microtine rodents [27]. Hence the costs of a mistake (recognition errors) differ between gray cuckoos and sparrowhawks on the one hand and rufous cuckoos and kestrels on the other. Consequently, the difference in host response could be due to differences in real danger and not plumage morph frequencies. According to this scenario, hosts should attack rufous cuckoos (mimicking the less dangerous model, the kestrel) more strongly than gray cuckoos (mimicking the more dangerous model, the sparrowhawk). However, we found the opposite.

Although our study provides the first experimental evidence that hosts can mistake the rufous cuckoo for a kestrel (host responses to both species were virtually identical), the functional significance of this resemblance remains unclear. Future studies are therefore needed to test whether similarities between the rufous cuckoo and kestrel is also a form of mimicry, like that of the sparrowhawk, or is only the result of the convergent evolution of cryptic plumage [11] to avoid detection by parasitized victims [7].

Finally, for such a study it will be crucial to use a consistent methodology and precise measures of host responses. As for the latter, we strongly recommend measuring host responses on continuous and not categorical scales. Honza et al. [5] assessed the most important component of great reed warbler defense, i.e., contact attacks, on a categorical scale (presence vs. absence of contact attacks; we consider contact attacks the most important component of host behavior because attacks can drive the laying female cuckoo away from a host nest or even kill it, (see above) whereas alarm calls cannot, see [28,29]). As we have shown in the present study (and in a previous study using different data sets: [16]), use of a categorical scale significantly diminishes variation in host responses and consequently lacks the power to detect even large existing biological effects (Table 1; see also Table one in [16]). Therefore, some existing differences might have gone undetected in the study of Honza et al. [5]. In general, the reduction of original continuous variation in host behavior into artificial categories is demonstrably misleading (Table 1) and should be avoided in future work (see also [18,21,22]).

## Conclusions

Payne [4] hypothesized that the “appearance of parasitic birds has been modified to avoid recognition by the hosts in the adult plumage and also in the eggs and nestlings”. How brood parasites escape host discrimination has been thoroughly studied at the egg stage (reviewed in [30]). Recent work also started to unravel parasite tricks that evolved as a response to host defenses at the nestling stage (reviewed in [30-32]). Nevertheless, our understanding of the co-evolutionary causes and consequences of brood parasite adult plumage variation is in its infancy [33]. Although dozens of studies have explored how hosts respond to adult cuckoos (e.g. [26,34,35]), the present study is the first to test, using consistent methodology, whether gray and rufous cuckoos do indeed mimic their hypothesized models (hawk and kestrel-mimicry hypotheses). Future studies may be extended to other exciting questions (see also [9,14]). How does the host aggression response and discrimination ability vary across multiple populations with varying frequencies of both models (predator species) and mimics (cuckoo morphs)? From the hosts’ perspective, is the rufous morph truly a better mimic of kestrels than the gray morph is of sparrowhawks? Do differences in adult plumage phenotype translate into other co-evolutionary adaptations and their effectiveness, e.g., egg mimicry?

## Materials and methods

### General field procedures

Field data were collected in a fishpond system near Štúrovo (47°51´N, 18°36´E, 115 m a.s.l.), south-western Slovakia mid May – mid July 2011–2012. For a detailed description of locality and all field procedures, including adult warbler mist-netting and color-ringing, nest searching and egg-marking, see [16].

Morph-ratio in the study area was established by counts of calling cuckoo females (following methods of Honza et al. [5]). Occurrence and frequency of predator models (sparrowhawks and kestrels) was determined by line transects throughout the entire study site. Both censuses were done during fieldwork in 2012.

In this research we followed guidelines of the Animal Behavior Society for the ethical use of animals in research. Licenses and permission to ring and handle the birds were provided by the Ministry of Environment of the Slovak Republic, No. 269/132/05-5.1pil and No. 7230/2008-2.1pil.

### Experimental procedures

We tested warbler responses to 5 different dummy (i.e., taxidermic stuffed birds) types: adult female cuckoo gray morph, adult female cuckoo rufous morph, adult male sparrowhawk, adult female kestrel, and adult turtle dove as a harmless control (hereafter: dove). Predator dummy sex was chosen to match size and color of respective cuckoo morphs [33]. For each dummy type we employed 2–4 specimens (depending on availability and destruction of already used dummies) which were chosen randomly for each experiment (see also [16] and references therein).

From 10 possible paired combinations of 5 dummies we excluded the combinations kestrel-sparrowhawk, kestrel-gray cuckoo and sparrowhawk-rufous cuckoo because those combinations are neither meaningful nor necessary for testing our hypotheses. The remaining 7 combinations were: turtle dove paired with each of the other dummies to test whether hosts recognize cuckoos and predators as special enemies; gray cuckoo-rufous cuckoo to test the hypothesis that warblers attacked the more common cuckoo morph more than the rarer cuckoo morph; kestrel-rufous cuckoo and sparrowhawk-gray cuckoo to test the perfection of rufous cuckoo- kestrel and gray cuckoo-sparrowhawk mimicry.

Previous work on the same study system used two different control dummies: turtle dove (in [15]) and collared dove (*S. decaocto*; in [16]). However, the selection of the control dummy did not affect results in our study system because responses (no. contact attacks min^−1^; mean ± S.E.) to both controls were statistically identical: turtle dove = 1.4 ± 0.7, n = 18; collared dove = 1.5 ± 0.7, n = 17; unequal variance t-test: t_32.78_ = 0.08, P = 0.93 (data for turtle dove from the present study). Further, responses to cuckoos paired with turtle vs. collared doves were also statistically identical: gray cuckoo paired with turtle dove = 31.9 ± 3.7, n = 18; gray cuckoo paired with collared dove = 32.8 ± 3.8, n = 17; unequal variance t-test: t_32.88_ = 0.17, P = 0.87. Thus, responses to different controls did not affect responses to cuckoo dummies paired with them and vice versa.

Following previous studies [15,16,36,37] we adopted a simultaneous presentation of dummy dyads at host nests (i.e., essentially a choice test). Great reed warblers attack almost all intruders near their nests, including the mounts of both cuckoo morphs and sparrowhawks at the highest levels of aggression [5,15,16,38-40]. Thus, successive presentation of single dummies would have very low power to detect any warbler discrimination abilities; therefore, only the simultaneous presentation of dummies enabled us to determine unambiguously the primary target of the host attack (see [15,16]). Compared to successive dummy presentation (e.g., [18]), the additional major advantage of this paired study design is that it eliminates risks of reinforcement or habituation [17,41], which would represent a serious problem because warblers in our study population show habituation during longer dummy presentations (see [16]). Therefore we set the duration of the experiment to a period before habituation commences, i.e., to 1 min. Further, the paired design automatically controls for many possible confounding factors that cannot be avoided by successive dummy presentations (see below).

Following already established protocols ([16] and references therein), two different dummies within a dyad were 0.8 m apart from each other, 0.5 m from the focal nest, at the same height above water level, and facing the nest rim. Within each experiment we randomized the side where each mount was located (i.e., left or right from observers’ direction).

At most nests (total n = 93), we presented only one experimental dyad. Because of the limited size of our study population and effects of predation we presented a second dyad at the same nest in some cases (n = 33) to reach representative sample sizes per experiment type. However, this does not represent a problem because (1) the second dyad was always a different combination of dummies than the first dyad, (2) the second experiment was done on the following day (cf. gap between trials with sequentially presented dummies was 15 min. or 2 h in other studies of predator mimicry, see [5] and [6] respectively) and (3) we controlled for repeated sampling by including nest id as a random effect in our statistical models (see below). Notably nest id explained an insignificant amount of variation (see below), which confirms that repeated testing of the subset of warbler pairs did not affect their responses.

Overall we tested 83 females and 84 males (breeding seasons 2011 and 2012 pooled). Out of these total numbers, only 10 females and 9 males were tested in both years of the study, all other individuals were tested in only one breeding season. This repeated sampling does not represent a problem because (1) it concerned only a minor part of the study population, (2) sampling of some individuals across a much shorter period (1 day within one breeding season) did not affect their responses (see above) making across year effects highly unlikely, and (3) we controlled for this repeated sampling of the same individuals statistically. As expected, individual id did not explain any amount of variation in our statistical models (see below), confirming that repeated testing of the small subset of females and males did not affect either their responses or our conclusions. We also note that none of the repeatedly tested females was paired to the same partner in both study breeding seasons.

Observations were made by the first author from a blind placed ~5 m from the focal nest. The other observer (1) simulated a human leaving the nest vicinity (i.e., two observers approached the nest, one entered a blind, the other left the area; this is a traditional method used by bird photographers and ringers to “persuade” nest owners there is no danger nearby the nest), and (2) helped to determine nest owners id (the other observer was ~10 m from the focal nest and had a chance to read the color rings both before and after the experiment; thus, we are confident our analyses are not confounded by presence of birds other than nest owners).

Each experiment started when the first contact attack by one of the nest owners at any of the two dummies occurred and lasted for 1 min. During all 126 experiments one or both of the dummies within a dyad were attacked, typically immediately after the arrival of nest owners. Forceful physical attacks may slightly change the position of the dummy and different postures could in principle affect host behavior (warblers seemed to increase their aggression after they tilted the dummy from its original position, AT unpubl. data). Therefore we excluded all experiments (n = 4) where a dummy did not remain in its original position throughout the whole experiment. Host aggression was measured as number of contact attacks per 1 min (for rationale behind excluding other potential measures see [16]).

### Statistical analyses

We followed analytical approaches of Trnka et al. [16] because they and the present study used identical experimental design. Differences in host responses to dummies within dyads were first tested with non-parametric Wilcoxon sign-rank tests. We then tested whether host responses were affected by confounding factors.

We avoided some potential confounders by experimental design; all dummy presentations were done on the first day of the incubation period, in the morning (7:00–11:00 CET), at non-parasitized nests, and only at nests of monogamous males, thus excluding nesting stage [42], daytime [41], parasitism status [39] and mating status [43] as confounders. We statistically controlled for other potentially relevant factors that could not be avoided in this study design, namely experiment date in the season (continual; including its squared term to test for non-linear seasonal patterns), clutch size (continual) as a surrogate of reproductive value [42], first attacking sex (categorical; male, female, both at once) and host arrival direction for the first arriving pair member (categorical; “central” = first host arrived directly at the nest with a chance to see both dummies simultaneously, “focal” = dummy side, i.e., host first saw the focal dummy and “opposite” = opposite dummy side, i.e., host first saw the dummy paired with the focal dummy). We only included arrival of the first pair member because the first member always started to respond before the second pair member arrived. Experiment date was centered around the mean within each year to exclude a possible confounding effect of between-year variation in our seasonal sampling effort (see also [35]). When clutch size was coded more conservatively as an ordinal variable the results remained the same (results not shown).

We included these variables plus the major factor of interest, dummy type (categorical), as predictors in the general linear mixed model (GLMM, normal error distribution, parameters estimated by REML, degrees of freedom estimated by Kenward-Roger method). Number of contact attacks per 1 min. was a continuous response. Response was not transformed as model residuals showed normal distribution as confirmed by visual inspection and Shapiro-Wilk tests. The paired nature of the experiment was modeled by pair id (as a nominal random effect, see below), and, therefore, a potential variable “experiment type” (Figure 1) was not necessary. Some other studies included first egg laying date in the clutch as another predictor (e.g., [35]), but this was neither necessary nor statistically meaningful as date of experiment was already included (note that a high correlation between date of experiment and first egg laying date would invalidate our statistical models due to multicollinearity, see [44]).

To control for potential year-, dummy specimen-, pair- and individual-related correlation in the data we included appropriate random effects. “Year” was entered as a random (i.e., not fixed) effect because we had no specific year-based temporal predictions (see also [35]). “Pair id”, “female id” and “male id” were entered as nominal random effects (note that 100% of tested individuals in the present study were of known identity due to being marked with color rings, see above). Both “female id” and “male id” were nested within “pair id” to reflect biological reality that the members of the same pair share the same territory.

In GLMM models, all random effects were negligible. Specifically, random effects explained from 0.00 to 3.31% of variation in the full, partially reduced or minimum adequate models, non-significantly different from 0 as assessed by their 95% CIs (i.e., confidence intervals overlapped 0 widely in all cases). Thus, there was no significant effect of year, dummy specimen, pair or individual on the variation in the data. Removal of these redundant random effects (as recommended by Bolker et al. [45]) also had no detectable effect on parameter estimates and, consequently, statistical significance and conclusions. Therefore, we decided to present statistical results without these random effects (following recommendations of [45]). Although nest id also explained little variation in host responses (3.31% of variation in the minimum adequate model, more than any other random effect) we kept it conservatively in all GLMM models. Nevertheless, removal of nest id had no effect on our conclusions (results not shown).

Coevolution between hosts and dangerous intruders near the nest may only increase pre-existing general “background” aggression, i.e., aggression to harmless intruders near the nest [18]. Therefore, “specific” aggression towards a dangerous intruder might be better measured as total observed aggression minus “background” aggression (see [18] for discussion and [5] for an example). Therefore, we performed analyses of both uncorrected total aggression (to make our results comparable with studies that used the same approach) and corrected specific aggression.

Although we performed multiple tests of responses to dummies, we did not apply a Bonferroni correction for two reasons. Generally, Bonferroni corrections are largely inappropriate for ecological studies (see [46] and references therein). Specifically, we did not test for any and all differences between dummies; instead we tested specific directional predictions of different hypotheses (see, e.g., Figure 1a vs. 1b vs. 1c).

Test statistics and p-values reported in Results for non-significant removed terms are from a sequential backward elimination procedure just before the particular term (being the least significant) was removed from the model. The final minimum adequate model contained, by definition, only significant predictors. We had specific *a priori* directional predictions, but the use of one-tailed tests in ecological studies is inappropriate [47]. Therefore, all tests in the present study are two-tailed. All analyses were done in JMP 8.0.1. (SAS Institute Inc., Cary, NC, USA).

## Competing interests

The authors declare that they have no competing interests.

## Authors’ contributions

AT conceived, designed and performed the experiments. TG analyzed the data and wrote the draft. Both authors read and approved the final manuscript.
